# Silica-Based Composite Sorbents for Heavy Metal Ions Removal from Aqueous Solutions

**DOI:** 10.3390/polym16213048

**Published:** 2024-10-30

**Authors:** Ramona Ciobanu, Florin Bucatariu, Marcela Mihai, Carmen Teodosiu

**Affiliations:** 1 Department of Environmental Engineering and Management, “Cristofor Simionescu” Faculty of Chemical Engineering and Environmental Protection, “Gheorghe Asachi” Technical University of Iasi, 73 D. Mangeron Street, 700050 Iasi, Romania; ramona.ciobanu@student.tuiasi.ro; 2 “Petru Poni” Institute of Macromolecular Chemistry, 41A Grigore Ghica Voda Alley, 700487 Iasi, Romania; fbucatariu@icmpp.ro

**Keywords:** core–shell composites, heavy metals, adsorption, wastewater treatment

## Abstract

Weak polyelectrolyte chains are versatile polymeric materials due to the large number of functional groups that can be used in different environmental applications. Herein, one weak polycation (polyethyleneimine, PEI) and two polyanions (poly(acrylic acid), PAA, and poly(sodium methacrylate), PMAA) were directly deposited through precipitation of an inter-polyelectrolyte coacervate onto the silica surface (IS), followed by glutaraldehyde (GA) crosslinking and extraction of polyanions chains. Four core–shell composites based on silica were synthesized and tested for adsorption of lead (Pb^2+^) and nickel (Ni^2+^) as model pollutants in batch sorption experiments on the laboratory scale. The sorbed/desorbed amounts depended on the crosslinking degree of the composite shell, as well as on the type of anionic polyelectrolyte. After multiple loading/release cycles of the heavy metal ions, the maximum sorption capacities were situated between 5–10 mg Pb^2+^/g composite and 1–6 mg Ni^2+^/g composite. The strong crosslinked composites (r = 1.0) exhibited higher amounts of heavy metal ions (Me^2+^) sorbed than the less crosslinked ones, with less PEI on the surface but with more flexible chains being more efficient than more PEI with less flexible chains. Core–shell composites based on silica and weak polyelectrolytes could act as sorbent materials, which may be used in water or wastewater treatment.

## 1. Introduction

Wastewater effluents originate from various industries and are polluted with nonbiodegradable organic/inorganic compounds and heavy metals. Among these contaminants, heavy metals (e.g., lead, nickel, zinc, arsenic, mercury, cadmium, chromium, copper, and iron) are harmful to human health and the environment. Usually, they are present in trace amounts, and due to their toxicity and nonbiodegradability, they can cause irreversible side effects in any form of life [1]. Furthermore, heavy metals are not only found as ions but can also combine with various organic pollutants in water, creating complex pollutants [2]. Often, these heavy metals exist as chemical complexes rather than as metal ions and are formed under a wide range of pH conditions [3]. Moreover, many other compounds in water, such as organic acids and antibiotics, can interact with heavy metals and generate combined compounds that can increase the complexity and difficulty of industrial wastewater treatment [4,5]. In addition, combined compounds can exhibit complex toxicological effects on enzymes, microorganisms, animals, and plants in the environment, and they can also contribute to the development of bacteria with complex resistance genes [6]. Consequently, it is essential to consider their combined effects rather than treating them in isolation.

However, heavy metals can have harmful effects on both living organisms and the environment, even in the form of ions, or in combination with other organic pollutants. For instance, Pb^2+^ is one of the most hazardous heavy metals [7] in aqueous media and has serious effects on cardiovascular diseases, lung problems, bone damage, and kidney, liver, and central nervous system disorders [8]. Another heavy metal with harmful effects is Ni^2+^, which includes, as the most common sources of human exposure, cigarette smoke, particulate matter of 2.5 μm (PM2.5), food, and drinking water [9]. According to the International Agency for Research on Cancer (IARC), Ni^2+^ belongs to the 1st category of agents that are carcinogenic to humans [10].

According to previously mentioned studies, it is of the utmost importance to mitigate these health risks and protect the well-being of humans and ecosystems by removing heavy metals from water and preserving environmental sustainability. Adsorption has gained popularity for the removal of heavy metals from aqueous solutions owing to its cost-effectiveness, high efficiency, and convenient operation [11]. Additionally, it has the potential to be used for a wide range of adsorbents from the well-known activated carbon [12], clay minerals [13], zeolites [14], biosorbents [15,16], and silica [17,18] to nanocomposites [19,20]. The latter has shown superior properties, such as higher adsorption capacity, large specific surface area, stability, selectivity, and reusability [21].

Recently, nanocomposites have received increased interest due to their numerous advantages in various applications. For instance, silica–polymer nanocomposites are promising, particularly silica–polyethyleneimine (SiO_2_-PEI), and can be efficiently used for odor removal [22], CO_2_ capture [23], and heavy metals removal from wastewater [24,25]. Silica is a low-cost ceramic, having excellent thermal properties. The surface characteristics are easily tunable because of their large surface area and mechanical stability [26]. Composite materials with a high adsorption capability for inorganic metals result from the combination of the flexibility and the abundance of the functional groups present in PEI. SiO_2_-PEI nanocomposites have been fabricated using different strategies [18]. For example, the removal of hexavalent chromium (Cr^6+^) from dilute aqueous solutions was achieved using iron-oxide-functionalized bio-based carbon–silica–polyethyleneimine composites. The composites were prepared by co-precipitation of Fe^2+^ and Fe^3+^ over Macadamia activated carbon, achieving a sorption capacity of 5.76 mg/g at pH = 1 [27]. In another study, two types of PEI-functionalized silica gels (SGs) were synthesized by single loading (s-PEI-SG) and dual loading (d-PEI-SG) for Cu^2+^ removal from multielement synthetic solutions. The d-PEI-SG reached a maximum Cu^2+^ adsorption capacity of 170.4 μmol/g, which was four times larger than s-PEI-SG (41.8 μmol/g) [28]. Meantime, Semenova et al. [20] designed a novel PEI-SiO_2_ nanocomposite with enhanced properties for Cu^2^⁺ adsorption by the deposition of SiO_2_ from organosilane tetraethoxysilane (TEOS) onto PEI microgel particles. A widely used technique for the modification of SiO_2_ microparticle surfaces with numerous functional groups of polyelectrolytes is the layer-by-layer (LbL) strategy. In one study, multilayer polyelectrolytes were deposited onto spherical silica using the LbL technique, creating nanostructured composites to adsorb metal ions (Cu^2^⁺, Pb^2^⁺, Ni^2^⁺, and Fe^2^⁺) from simulated and real surface waters [24].

Nevertheless, these methods have their own limitations, such as a relatively low efficiency, requirement of organic solvents, necessity of a heating source or electricity, use of complex and time-consuming synthesis processes, and production of toxic slurries [28,29]. However, the urge to synthesize composites with a high sorption capacity and selectivity toward different heavy metal ions has made one-pot synthesis a promising method for many benefits, such as simplicity of operation, time savings via reduction of the intermediate steps in purification, high efficiency, and economy. Another advantage of one-pot synthesis is that it occurs under ambient conditions and aligns with the principles of sustainable chemistry by reducing waste and energy consumption [30,31]. A few studies have reported the successful use of a one-pot strategy in the synthesis of SiO_2_-PEI nanocomposites for heavy metal ions removal from aqueous solutions. A PEI/sodium silicate composite was synthesized using poly(ethylene glycol) diglycidyl ether as a crosslinking agent. The authors achieved a Langmuir sorption capacity of 137 mg/g for Cu^2+^ [31]. Similarly, Choi et al. [32] investigated the removal efficiency and mechanism of Cr^3+^ using PEI–silica nanoparticles, achieving a high adsorption capacity of 183.7 mg/g. Pb^2^⁺ exhibited a maximum sorption capacity of 125.68 mg/g with a composite sorbent with a silica core and polyelectrolyte coacervate shell synthesized via a one-pot technique.

The research objectives of this study refer to the (a) examination of the influence of the type of polyanion involved in the formation of the polyelectrolyte complex in the direct deposition step; (b) influence of the crosslinking degree of the composite shell on the amount of heavy metal ions (Pb^2+^ and Ni^2+^) sorbed; (c) identification of equilibrium models for the sorption process; and (d) determination of the regeneration capacity of silica composite sorbents.

The novelty of this study concerns the determination of the maximum sorption capacity of four silica-based composites obtained through coacervate deposition to remove Pb^2+^ and Ni^2+^ ions from aqueous solution. Furthermore, the possible adsorption mechanisms were explored and kinetic models, equilibrium isotherms, and sorbent regeneration were investigated in a methodical manner.

## 2. Materials and Methods

### 2.1. Chemicals

In this study, all chemical reagents were of analytical grade and purchased as follows: lead nitrate—Pb(NO_3_)_2_—and nickel nitrate—Ni(NO_3_)_2_—from Fluka (Darmstadt, Germany), and nitric acid, hydrochloric acid, and sodium hydroxide from Merck, Germany. Silica microparticles (ISs) with diameters in the range of 40–60 microns were purchased from Daiso Chemical Co. (Osaka, Japan). Poly(sodium methacrylate) (PMAA) (Mw = 1800 g/mol), poly(acrylic acid) (PAA) (Mw = 10,000 g/mol), branched poly(ethyleneimine) (PEI) (Mw = 25,000 g/mol), and crosslinking agent glutaraldehyde (GA) were purchased from Sigma-Aldrich (Merck, Darmstadt, Germany).

All chemicals were used as received without further purification. Ultrapure water was used to prepare all solutions throughout the study.

### 2.2. Synthesis of the Silica Composites

The synthesis procedure has been detailed in a previous study [25]. A brief description of the silica composite synthesis used in this study is provided below.

- ISs (5.0 g) were suspended in 12 mL of 1 M PEI, followed by the gradual addition of 6 mL of either PAA or PMAA at the same concentration under energetic stirring conditions until the solution became transparent;
- Crosslinking with GA at two different molar ratios (r), namely, a weak degree, r = [—CHO]:[—NH_2_] = 1:10, and a strong degree, r = [—CHO]:[—NH_2_] = 1:1. Based on the content of the precipitated PEI, the volume of GA (2.5% *w*/*v*) required for the crosslinking of each sample was calculated;
- Extraction of the non-crosslinked polyelectrolyte chains from the silica composite microparticles was carried out with a strong basic (1 M NaOH) or acidic (1 M HCl) treatment to ensure the flexibility of the organic shell.

In this way, by using two different polyanions (PAA and PMAA), combined with a weak (r = 0.1) and a strong (r = 1.0) PEI crosslinking degree, four types of polymeric shells were generated around the inorganic core, as follows: IS/(PEI/PAA)_r=0.1_; IS/(PEI/PAA)_r=1.0_; IS/(PEI/PMAA)_r=0.1_; and IS/(PEI/PMAA)_r=1.0_.

The synthesis, characterization [25], and dynamic sorption tests of these composites [33] have been published. To determine the maximum sorption capacity of these composites, we highlight the behavior of Pb^2+^ and Ni^2+^ ions in a single adsorption system, having previously studied Pb^2+^ and Ni^2+^ ions in competition with Cd^2+^ ions [25,33]. In this study, batch sorption studies of four silica composites for the removal of Pb^2+^ and Ni^2+^ from aqueous solutions were performed.

### 2.3. Batch Sorption and Desorption Studies

**Figure 1:**
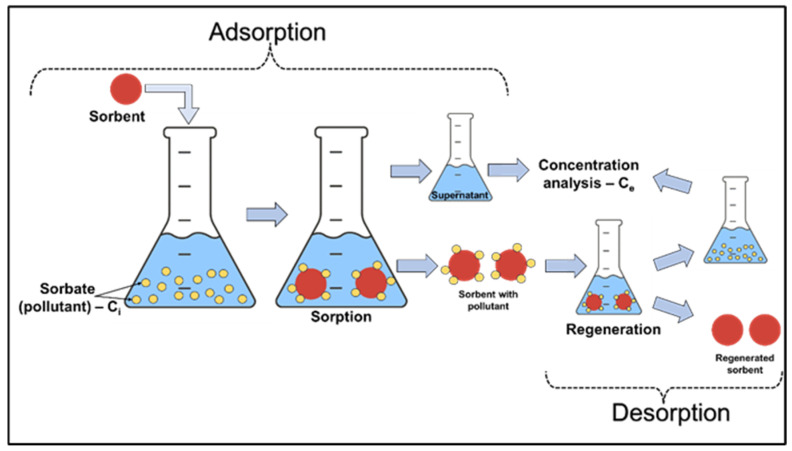
Schematic illustration of the batch sorption/desorption.

Batch experiments were carried out on the laboratory scale in 20 mL closed vials, containing a certain amount of silica composite sorbents with 10 mL of various concentrations of Pb^2+^ and Ni^2+^ solutions, for 3 h at room temperature under stirring conditions (Table 1). Stock solutions containing Pb(NO_3_)_2_ and Ni(NO_3_)_2_, each with a concentration of 5 mM, were first prepared. Afterwards, different aqueous solutions were prepared from the stock solution dilution to obtain solutions of Me^2+^ ions with different initial concentrations for the batch equilibrium studies. At the end of the contact time, supernatants were collected to evaluate the performance of the sorbents and their removal efficiencies. The desorption study included the same conditions under which the batch adsorption experiments on the laboratory scale were performed (Figure 1).

The effect of the adsorption time on the adsorption performances was investigated only for Pb^2+^ uptake solution in time ranges from 1 to 24 h, at a fixed 100 mg/L of the initial Pb^2+^ concentration and 100 mg of the adsorbent dosage.

After adsorption, the silica composite sorbents loaded with the heavy metal ions were washed with 10 mL ultrapure water. Subsequently, 10 mL of HNO_3_ (1 M) were used to extract the metal ions for 1 h. The composite materials were then three times washed with 10 mL ultrapure water until the initial pH of ultrapure water was reached. Before another sorption cycle, the composite materials were activated with 1 mL of NaOH (1 M) and washed to neutral pH with distilled water [25]. This sorption–desorption cycle was performed three times for each composite.

The eluates were analyzed for the heavy metal concentrations using atomic absorption spectroscopy (contrAA 800, Analytik Jena, Jena, Germany). Before analysis, 1 mL of 0.5% nitric acid was added to each sample to ensure the stability of the heavy metals in the solution, as many of them can hydrolyze and form hydroxides or oxides that are poorly soluble in water. The amount of metal ion adsorbed per gram of composite, q_e_, and the adsorption removal efficiency, RE (%), were determined with the following equations [25]:

(1) $$q_{e}=\left(C_{i}-C_{e}\right)(\frac{V_{e}}{m})$$

(2) $$RE=\frac{C_{i}-C_{e}}{C_{i}}\times 100$$

where q_e_ (mg/g of composite) is the adsorption capacity at equilibrium, C_i_ (mg/L) is the initial heavy metal concentration, C_e_ (mg/L) is the heavy metal concentration at equilibrium, V_e_ (L) is the heavy metal ion solution volume, and m (g) is the amount of silica composite sorbent.

### 2.4. Adsorption Data Modeling

The identification of equilibrium models is critical for the sorption process, particularly for the calculation of the maximum sorption capacities derived from these models. In this study, the equilibrium data obtained from the influence of the initial concentration studies were used for the modeling of several isotherms, namely, Langmuir, Freundlich, Sips, and Toth [34,35]. To describe the relationship between the adsorption capacities of the composite sorbents and the contact time, the pseudo-first-order (PFO) model and pseudo-second-order model (PSO) were employed to fit the kinetic data [36]. To determine the isotherm and kinetic models that best fit the experimental data, we used the following four error functions: sum of the square errors, chi-square test, hybrid fractional error function, and average relative error. After nonlinear regression optimization, the methodology of the sum of normalized errors (SNE) was applied by using the “Solver add-in” from Microsoft Office Excel, which facilitated the selection of the optimum set of parameters for each isotherm [37]. The lowest SNE value indicated the optimal set of parameters for a specific model. Model adequacy was determined using the Akaike information criterion (AIC) [37,38]. This criterion is based on the maximum likelihood theory and reduces the overfitting caused by models with more parameters. Table 2 lists all equations used in modeling the adsorption data.

## 3. Results and Discussion

### 3.1. Adsorption Isotherms

**Figure 2:**
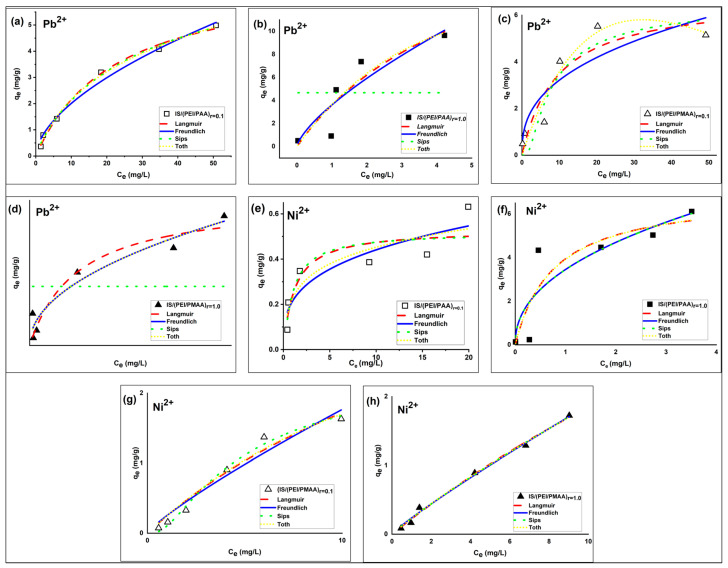
Nonlinear isotherms of Pb^2+^ (**a**–**d**) and Ni^2+^ (**e**–**h**) on IS/(PEI/PAA) and IS/(PEI/PMAA) silica composite sorbents (C_i_ Pb^2+^ = 5–100 mg/L, C_i_ Ni^2+^ = 1–26 mg/L, m_sorbent_ = 100 mg, temperature ~22 °C, contact time = 3 h).

The IS/(PEI/PAA) and IS/(PEI/PMAA) composite sorbents were tested for Pb^2+^ and Ni^2+^ ions removal from aqueous solutions. The relationship between the sorption capacity of the silica composite sorbents and the heavy metal ions concentration of the aqueous phase at equilibrium can be observed in Figure 2.

The sorption performances of the silica composite materials for Pb^2+^ and Ni^2+^ ions were assessed by building and modeling equations with two and three parameters, respectively, of the adsorption isotherms. The best-fit parameters obtained from the Langmuir, Freundlich, Sips, and Toth isotherms (in bold) and error analysis results are presented in Table 3.

The optimal parameters given in Table 3 were determined based on the SNE method for each composite sorbent used in the sorption processes. Among the error functions, the Chi-square test provided the optimum isotherm parameters in most cases. The Akaike information criterion (AIC) was used to rank the adsorption isotherms. According to the definition of AIC, the smallest AIC value indicates the most suitable model for describing the experimental data, even if the values are negative [39]. For the Pb^2+^ ion sorption processes, the IS/(PEI/PAA)_r=1.0_ sorbent had the smallest AIC value (i.e., −5.48) of the Freundlich isotherm, closely followed by the Toth model, followed by the Sips and Langmuir isotherms. In addition, the same sorbent obtained a minimum AIC value (i.e., −5.11) corresponding to the Freundlich isotherm for Ni^2+^ ion sorption studies. When the IS/(PEI/PMAA)_r=1.0_ sorbent was used, the isotherms were ranked as follows: Toth < Langmuir < Sips < Freundlich for Pb^2+^ ion sorption processes and Freundlich < Sips < Toth < Langmuir for Ni^2+^ ion sorption studies. In general, the strong crosslinked composite materials own the smallest AIC values.

The Freundlich isotherm equation provided the best approximation of experimental data for most composite sorbents. The Freundlich isotherm can be used to define multilayer adsorption on heterogeneous surfaces [35]. The exponent of the Freundlich isotherm, 1/n_F_, indicates the intensity of adsorption and is usually greater than zero (0 < 1/n_F_ < 1). When 1/n_F_ is greater than 1, the adsorption process is unfavorable and irreversible when 1/n_F_ = 1 [40]. The values of 1/n_F_ obtained in this study show that heterogeneous adsorption of Pb^2+^ and Ni^2+^ ions occur in most composite sorbents. The IS/(PEI/PAA)_r=1.0_ achieved a value greater than unity, indicating an unfavorable adsorption process, and IS/(PEI/PMAA)_r=0_._1_ obtained 1/n_F_ = 1. The irreversibility of the isotherm can be attributed to the fact that the concentration should be very low before desorption.

**Figure 3:**
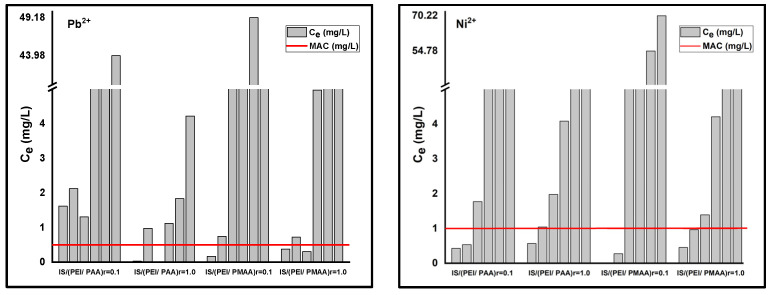
Maximum allowed concentrations (MACs) of Pb^2+^ and Ni^2+^ in wastewaters and the final concentrations of adsorption studies (C_e_) for each composite material.

Furthermore, lower values of this parameter, as in the case of most composite materials, suggest the presence of systems with increased heterogeneity. According to Freundlich isotherm, the maximum sorption capacities for the composite sorbents are 5.258 mg Pb^2+^/g composite and 0.54 mg Ni^2+^/g composite for IS/(PEI/PAA)_r=0.1_, 10.39 mg Pb^2+^/g composite and 6.96 mg Ni^2+^/g composite for IS/(PEI/PAA)_r=1.0_, 5.81 mg Pb_2+_/g composite and 1.85 mg Ni^2+^/g composite for IS/(PEI/PMAA)_r=0.1_, and 7.9 mg Pb^2+^/g composite and 1.74 mg Ni^2+^/g composite for IS/(PEI/PMAA)_r=1.0_. The maximum sorption capacities estimated using this model were close to the maximum amount of metal ions adsorbed on each composite sorbent. The maximum sorption capacities have been situated between 5–10 mg Pb^2+^/g composite and 1–6 mg Ni^2+^/g composite. The final concentrations (C_e_) were found to be lower than Romanian maximum allowed concentrations (MACs) of effluent discharge in receiving waters [41] for almost all composite materials at the lowest initial concentrations. For example, at the lowest tested initial concentration (i.e., 1 mg/L), Ni^2+^ removal was very efficient for all composite materials. In case of Pb^2+^ removal, only the composites sorbents made from PEI/PMAA had the final concentration below MAC in the effluent (i.e., 0.5 mg/L). Figure 3 presents a comparison between the MACs of lead and nickel in wastewaters and the final concentrations of the adsorption studies.

The isotherm that showed a better fit was the Toth model. The Toth model is derived from the Langmuir isotherm model and can be applied to heterogeneous sorption processes [40]. Its exponent n_T_ indicates the surface heterogeneity and when n_T_ = 1, the adsorption process is considered homogeneous. The value of n_T_ was higher than unity for most of the composite materials suggesting the heterogeneity of the system in the case of both metal ions uptake.

At the same time, it can be observed the maximum sorption capacities obtained were dependent on the number of functional groups provided by the crosslinking ratio. Thus, the strongly crosslinked composite exhibited higher sorption capacities than the weakly crosslinked composite. The availability of functional groups is also influenced by the polyanion used (PAA or PMAA), which is given by the packing mode (coil or linear), and by its nature further influences the sorption mechanism.

**Figure 4:**
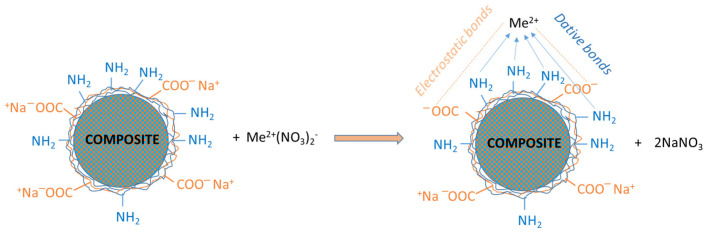
Principal types of interactions among heavy metal ions (Me^2+^) with composite surfaces activated with NaOH 1 M.

The sorption process of heavy metal ions (Me^2+^) on these core–shell composite microparticles is mainly driven by coordination bonds and ion exchange, as shown in Figure 4.

An ideal equilibrium between these interactions is required for an effective sorption process; thus, all functional groups (amino and carboxyl) had been activated before every immobilization process. This activation was accomplished by using an aqueous solution of NaOH. The heavy metals salt could interact electrostatically with anionic compounds. The formation of dative bonds may involve amino groups, because of the free “p” electrons present in the nitrogen atom. In conclusion, coordinative/ionic bonds were formed between heavy metal ions and any functional groups present on the polyelectrolyte shell.

### 3.2. Influence of Contact Time and Kinetics

**Figure 5:**
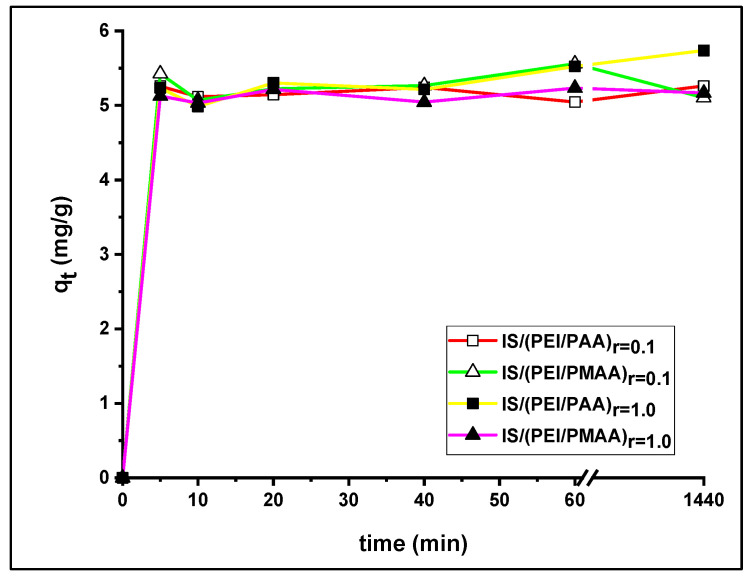
Influence of contact time on Pb^2+^ ions adsorption.

The influence of contact time on Pb^2+^ uptake over 24 h is shown in Figure 5. All profiles were very similar, characterized by a steep slope at the start of the reaction time. In the first 10 min, almost the same amount at equilibrium was achieved for both the weakly reticulated composite sorbents and the composite sorbents with a higher crosslinking ratio. The uptake capacity steadily increased until 60 min for all composite materials, followed by a small change in the uptake amount until equilibrium was reached.

Adsorption kinetics modeling allows the estimation of the sorption rate constant and provides insight into possible reaction mechanisms. The kinetic parameter values of the pseudo-first- and pseudo-second-order models obtained following the SNE procedure are summarized in Table 4.

The lowest AIC values were found for the pseudo-first-order model for the majority of the composite sorbents, with the exception of IS/(PEI/PMAA)_r=0.1_. For the latter, the PSO model closely followed, with an insignificant difference. For all sorbents, the q_e_ values evaluated using the PFO model were in very good agreement with the equilibrium capacities. These results are also supported by the model’s selection criteria. The PFO’s parameter k_1_ describes how fast adsorption equilibrium is achieved. A small value of k_1_ and a large value of (q_e_, q_t_) indicate that the adsorption processes are slow.

### 3.3. Sorbent Regeneration/Reusability

**Figure 6:**
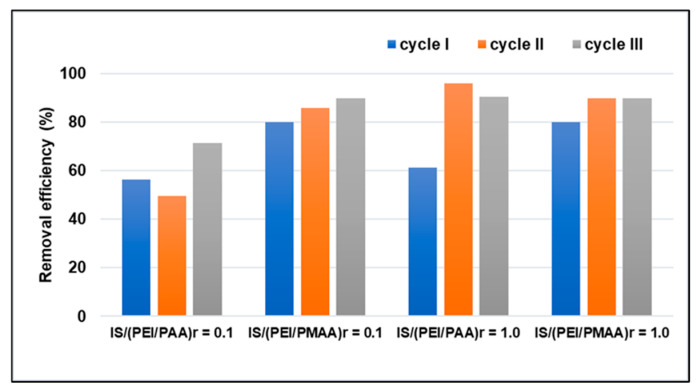
Consecutive adsorption–desorption cycles of silica composite sorbents for Pb^2+^ removal (C_i_ = 100 mg/L).

Another important characteristic of a good practical sorbent is its regeneration capacity, which is why the potential for reuse of the silica composite sorbents was investigated. Desorption of heavy metals was carried out using HNO_3_ (1 M). Before the sorption experiments, each composite sample was treated at an extreme pH, with a strong base to extract polyanions and strong acids to remove the non-crosslinked polycationic chains. Therefore, the composite shell was stable during multiple pollutant sorption (a wide range of pH) and regeneration processes. The Pb^2+^ removal efficiencies of the four silica composite sorbents after three adsorption–desorption cycles are presented in Figure 6.

From the results illustrated in Figure 6, it can be observed that the fresh material subjected to the first sorption cycle had a lower capacity than that of the other two cycles. A possible reason could be that after several cycles of sorption–desorption, the structural network of the material becomes flexible so that the pollutant can easily penetrate its depth, and, in this way, the sorption takes place through better diffusion in the material.

Based on these results, we can conclude that all four tested composite sorbents exhibited good cycle regeneration and stability, indicating their practical application in wastewater treatment. Furthermore, it is possible to remove the soft shell from the spent polyelectrolyte composite materials, thereby facilitating the recovery and reuse of the inorganic core. However, further experiments are needed that consider a higher number of cycles, since this would help define the applicability of the sorbent materials and improve the quality of the results for these composite materials.

## 4. Conclusions

Heavy metal ions sorption tests were carried out on silica composites obtained by the one-pot coacervate deposition method, which showed that the amount of adsorbed Pb^2+^ and Ni^2+^ drastically depended on the organic content given by the crosslinking degree. The silica composite sorbents achieved maximum sorption capacities, between 5 and 10 mg/g composite for Pb^2+^ and 1 to 6 mg/g composite for Ni^2+^.

The strong crosslinked composites (r = 1.0) exhibited higher amounts of sorbed Me^2+^ than the less crosslinked ones. Thus, less PEI on the surface but with more flexible chains is more efficient than more PEI with less flexible chains. The amounts of immobilized Me^2+^ depended on the type of anionic polyelectrolyte, with the PMAA composites obtaining slightly higher amounts of sorbed/desorbed Me^2+^ compared to the PAA composites.

The equilibrium and kinetics data were assessed in a methodical manner to determine the optimum model parameters and to rank the models. According to the model evaluation, the Freundlich isotherm model and pseudo-first-order model best described the sorption process for most of the silica composite sorbents. The PFO model represents adsorption diffusion, and the Freundlich model describes the physical adsorption processes that underlie the adsorption mechanism in this study.

The three sorption/desorption cycles of Pb^2+^ ions demonstrated the possibility of using silica composites in wastewater treatment. However, such a possibility should be studied under dynamic conditions, because such sorbents should be deposited in sorption columns to enable industrial operation.

This study demonstrates that use of the one-pot coacervate approach can result in versatile composite materials with applications in solid-phase extraction, wastewater treatment, and selectivity studies on the targeted pollutants.

## Figures and Tables

**Table 1:** Experimental conditions of sorption studies.

Composite Sorbent	Crosslinking Degree (r)	Amount of Composites (mg)	Contact Time (h)	C_i_ Pb^2+^ (mg/L)	C_i_ Ni^2+^ (mg/L)
IS/(PEI/PAA)	r = 0.1	100	3	5; 10; 20; 50; 75; 100	1; 2; 5; 13; 19; 26
	r = 1.0				
IS/(PEI/PMAA)	r = 0.1				
	r = 1.0				

**Table 2:** Equations for the isotherm and kinetic models and error functions used in the adsorption data modeling.

Model	Equation	Equation No.
Isotherm models		
Langmuir	$q_{e}=\frac{q_{m,L}K_{L}C_{e}}{1+K_{L}C_{e}}$	(3)
Freundlich	$q_{e}=K_{F}C_{e}^{1/n_{F}}$	(4)
Sips	$q_{e}=\frac{q_{m,S}K_{S}{C_{e}}^{n_{S}}}{1+K_{S}{C_{e}}^{n_{S}}}$	(5)
Toth	$q_{e}=\frac{q_{m,T}K_{T}C_{e}}{{\left(1+{\left(K_{T}C_{e}\right)}^{n_{T}}\right)}^{1/n_{T}}}$	(6)
Kinetic models		
Pseudo-first-order	$q_{t}=q_{e,1}(1-\exp\left(-k_{1}t\right))$	(7)
Pseudo-second-order	$q_{t}=\frac{k_{2}q_{e,2}^{2}t}{1+k_{2}q_{e,2}t}$	(8)
Error functions		
Sum of the square error (SSE)	$\sum_{j=1}^{x}{\left(q_{j,exp}-q_{j,model}\right)}^{2}$	(9)
Chi-square test (χ^2^)	$\frac{\sum_{j=1}^{x}{\left(q_{j,exp}-q_{j,model}\right)}^{2}}{q_{i,exp}}$	(10)
Derivative of hybrid fractional error function (HYBRID)	$\frac{100}{x-p}\sum_{j=1}^{x}{\left(q_{j,exp}-q_{j,model}\right)}^{2}$	(11)
Average relative error (ARE)	$\frac{100}{x}\sum_{j=1}^{x}\left|\frac{q_{j,exp}-q_{j,model}}{q_{j,exp}}\right|$	(12)
Akaike information criterion (AIC)	$2p-x\left[\ln\left(\frac{SSE}{\left(x-p\right)}\right)\right]$	(13)

q_m,i_—The maximum adsorption given by the respective model; K_i_—the constant of isotherm i; k_i_—the constant of kinetic model i; n_i_—the exponent of model i; a—the initial sorption rate; b—the desorption constant; t—time; q_exp_—the experimental adsorption capacity; q_model_—the adsorption capacity calculated from the model; x—the number of data points; and p—the number of parameters in the model.

**Table 3:** The best-fit isotherm parameters of silica composite for removal of Pb^2+^ and Ni^2+^ (C_i_ Pb^2+^ = 5–100 mg/L; C_i_ Ni^2+^ = 1–26 mg/L; m_sorbent_ = 100 mg; temperature = ~22 °C; contact time = 3 h).

Isotherm Model	Parameters	Pb^2+^				Ni^2+^			
		IS/(PEI/PAA)_r=0.1_	IS/(PEI/PAA)_r=1.0_	IS/(PEI/PMAA)_r=0.1_	IS/(PEI/PMAA)_r=1.0_	IS/(PEI/PAA)_r=0.1_	IS/(PEI/PAA)_r=1.0_	IS/(PEI/PMAA)_r=0.1_	IS/(PEI/PMAA)_r=1.0_
Langmuir	q_m,L_ (mg/g)	**7.050**	**22.08**	**7.161**	**9.156**	**0.518**	**30.122**	**21.77**	**12.638**
	K_L_ (L/mg)	**0.043**	**0.195**	**0.076**	**0.188**	**0.613**	**0.076**	**0.008**	**0.017**
	SSE	0.096	10.45	3.54	3.36	0.04	12.21	0.12	0.01
	χ^2^	0.07	8.44	1.62	1.34	0.10	3.56	0.12	0.04
	HYBRID	2.34	301.74	36.27	17.64	8.52	109.34	10.52	3.73
	ARE	8.60	81.53	41.94	24.47	22.29	61.18	22.79	12.27
	AIC	26.37	−2.24	4.73	5.05	31.48	−2.69	25.23	38.42
Freundlich	K_F_	**0.422**	**2.006**	**1.00**	**1.713**	**0.185**	**1.384**	**0.177**	**0.211**
	1/n_F_	**0.642**	**1.142**	**0.452**	**0.733**	**0.359**	**1.287**	**1.00**	**0.958**
	SSE	0.336	19.98	5.15	3.28	0.03	18.28	0.15	0.02
	χ^2^	0.185	4.63	1.39	1.39	0.11	4.28	0.13	0.04
	HYBRID	6.87	67.29	13.46	24.28	14.35	48.49	6.40	3.81
	ARE	14.98	52.48	24.01	31.15	26.35	42.66	18.80	12.66
	AIC	18.86	−5.48	2.49	5.20	32.54	−5.11	23.81	37.36
Sips	q_m,S_	**7.45**	**7.31**	**3.01**	**10.01**	**0.31**	**5.03**	**2.384**	**4.63**
	K_s_	**0.045**	**9.98**	**15.97**	**0.146**	**1.587**	**13,625**	**0.07**	**0.05**
	n_S_	**0.95**	**202.38**	**100.21**	**0.98**	**1.66**	**9.93**	**1.52**	**1.13**
	SSE	0.08	11.40	15.55	3.48	0.03	1.49	0.04	0.02
	χ^2^	0.07	2.22	5.49	1.38	0.06	0.39	0.03	0.03
	HYBRID	3.52	64.72	123.48	21.80	5.27	34.90	1.71	3.42
	ARE	9.49	34.70	70.76	20.80	14.49	21.74	7.17	9.45
	AIC	27.94	−2.70	−3.87	5.10	34.43	10.18	32.58	37.68
Toth	q_m,T_	**8.30**	**22.62**	**320.03**	**9.27**	**0.44**	**6.22**	**1.63**	**3.59**
	K_T_	**0.04**	**0.1**	**0.578**	**0.16**	**0.55**	**0.368**	**0.12**	**0.06**
	n_T_	**0.82**	**100.14**	**0.125**	**1.05**	**3.16**	**4.77**	**31.55**	**1.97**
	SSE	0.08	17.84	4.71	3.54	0.05	11.50	0.04	0.01
	χ^2^	0.07	4.80	1.39	1.38	0.10	3.44	0.08	0.04
	HYBRID	3.51	161.80	21.23	21.81	9.64	150.41	16.50	4.61
	ARE	9.51	64.58	27.54	27.72	17.71	61.06	22.01	11.86
	AIC	27.76	−4.94	3.28	5.01	31.06	−2.06	31.41	38.14
Experimental	q_exp_ (mg/g)	**4.98**	**9.63**	**5.52**	**8.03**	**0.63**	**2.62**	**1.63**	**1.72**

Note: The best-fit parameters obtained from the Langmuir, Freundlich, Sips, Toth isotherms and experimental adsorption capacities are presented in bold.

**Table 4:** Kinetic parameters obtained for the adsorption of Pb^2+^ ions onto composite sorbents.

Model	Parameters	Pb^2+^			
		IS/(PEI/PAA) _r=0.1_	IS/(PEI/PAA) _r=1.0_	IS/(PEI/PMAA) _r=0.1_	IS/(PEI/PMAA) _r=1.0_
PFO	k_1_ (1/min)	**3.88 × 10^5^**	**0.74**	**26.96**	**1.19**
	q_e,1_ (mg/g)	**51.44**	**53.45**	**52.63**	**51.39**
	SSE	4.69	33.20	17.87	3.62
	χ^2^	0.09	0.61	0.33	0.07
	HYBRID	0.04	0.28	0.15	0.03
	ARE	1.46	3.47	2.68	1.31
	AIC	3.043	−8.69	−4.98	4.58
PSO	k_2_ (g/(mg⋅min))	**1.00 × 10^30^**	**0.04**	**1.00 × 10^30^**	**0.30**
	q_e,2_ (mg/g)	**51.76**	**54.64**	**52.69**	**51.57**
	SSE	3.99	22.87	17.81	3.32
	χ^2^	0.08	0.43	0.33	0.06
	HYBRID	0.04	0.20	0.15	0.03
	ARE	1.47	3.34	2.70	1.3
	AIC	4.00	−6.46	−4.96	5.10
Experimental	q_exp_ (mg/g)	**52.62**	**57.38**	**52.7**	**51.6**

Note: Kinetic parameters and experimental adsorption capacities are presented in bold.

## Data Availability

The original contributions presented in the study are included in the article, further inquiries can be directed to the first author.
